# Supplementary material for: Continuous Synthesis of Spherical Polyelectrolyte Brushes by Photo-Emulsion Polymerization in a Microreactor
Source: Polymers (Basel). 2023 Nov 30;15(23):4576. doi: 10.3390/polym15234576 (PMC10708043; doi:10.3390/polym15234576)
Supplement: Supplementary file 1 [file polymers-15-04576-s001.zip › polymers-2698156-supplementary.pdf]

Supplementary Materials

Continuous synthesis of Spherical polyelectrolyte brushes by photo-emulsion polymerization in Microreactor

Ziyu Zhang [1], Yuhua Zhang [1], Yang Tian [1], Zhinan Fu [1], Jiangtao Guo [1], Guofeng He[1,2], Li Li[1], Fang Zhao[1], and Xuhong Guo [1,3,*]

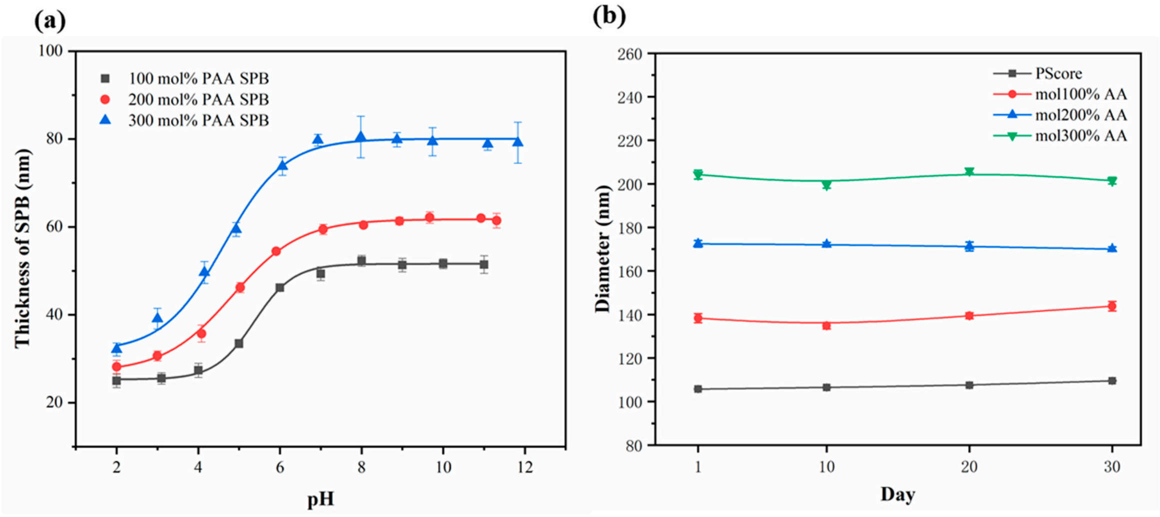

Figure S1. Particle size at different molar amounts of monomers in (a) pH responsiveness (b) stability.

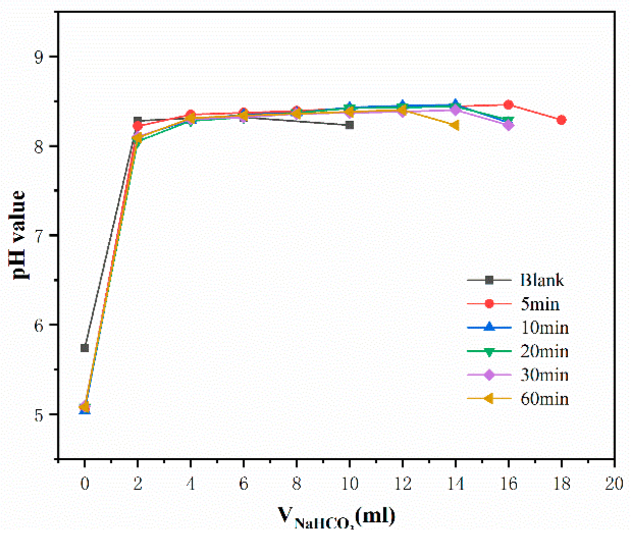

Figure S2. pH variation of the solution with the addition of PS-PAA SPB scale inhibitor in different residence time.

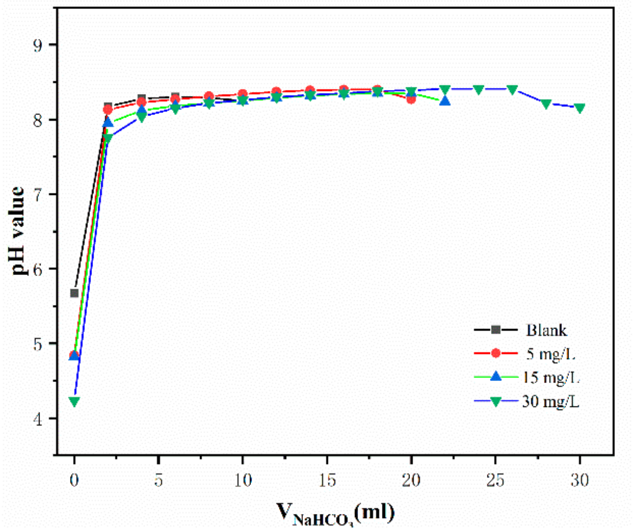

Figure S3. pH variation of the solution with the addition of PS-PAA SPB scale inhibitor in different dosage.

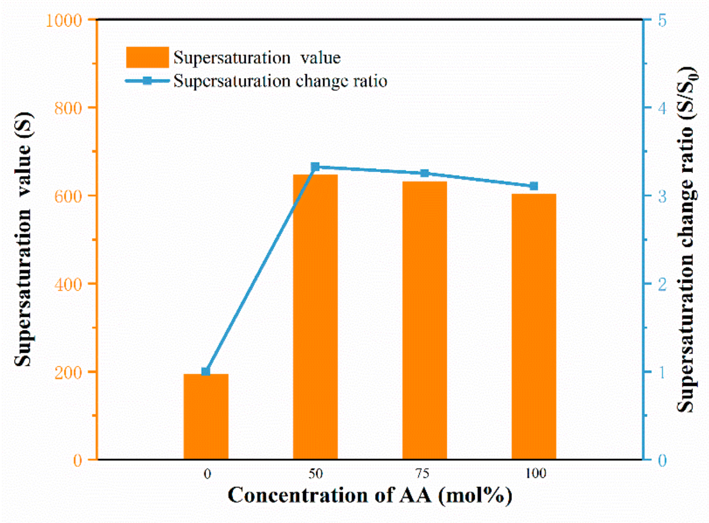

Figure S4. Variation of calcium carbonate supersaturation on the effect of concentration of AA.
